# Contribution of pulse oximetry in relation to respiratory ﬂow events in a home-based approach aimed at diagnosing obstructive sleep apnea

**DOI:** 10.5935/1984-0063.20200042

**Published:** 2021

**Authors:** Eduardo Enrique Borsini, Magali Blanco, Glenda Ernst, Alejandro Salvado, Ignacio Bledel, Carlos Alberto Nigro

**Affiliations:** 1 Hospital Británico de Buenos Aires, Sleep - Buenos Aires - Capital Federal - Argentina.; 2 Hospital Alemán de Buenos Aires, Sleep - Buenos Aires - Capital Federal - Argentina.

**Keywords:** Oximetry, Diagnosis, Sleep Apnea Syndrome

## Abstract

**Objective:**

To compare pulse oximetry with manual analysis against all signals of respiratory polygraphy.

**Material and Methods:**

This retrospective study estimated sensitivity (S), specificity (Sp) and positive/negative likelihood ratio (LR+/-) of the oxygen desaturation index (ODI-test) and apnea-hypopnea index (AHI-reference).

**Results:**

3854 patients (61.5% men) were included. Age, BMI, Epworth sleepiness scale and AHI were: 55 years (44-65), 30.9kg/m^2^ (27-36), 7 points (4-11), and 14 events/hour (6-25), respectively. 18% showed an AHI <5 events/hour, 34% = 5 and <15, 27% = or > 15 and < 30, and 31% > 30. The S, Sp, and LR+/- of ODI for AHI = 5 events/hour was 93%, 92%, 12 and 0.08 with an accuracy of 93%. For AHI = 15 events/hour, the values were: S 94%, Sp 94%, LR+ 15 and LR- 0.06 and 94% accuracy (r^(2)^ Spearman: 0.92).

**Conclusion:**

In a population at a high risk for OSA, home-based pulse oximetry had a diagnostic accuracy > 90% when is compared against all respiratory signals obtained from simpliﬁed home sleep testing.

## INTRODUCTION

Obstructive sleep apnea (OSA) is an emerging public health issue due to its high prevalence and morbimortality as well as its potential to develop car accidents, domestic and work-related accidents and cardiovascular complications^1-4^.

OSA has become an emerging health issue due to its high prevalence. Considering a diagnostic criterion based on an apnea-hypopnea index (AHI) >5 events per hour, it affects >18% of men and >10% of women in the general adult population^5^. Consequently, the scientific community needs to find pragmatic strategies to evaluate one billion people suffering from this sleep disorder worldwide^6^.

Although OSA diagnosis has traditionally been confirmed by a polysomnography performed at a sleep laboratory, validated respiratory polygraphy (RP) are also accepted in populations with a low/high likelihood of suffering from this disease^7-9^. RP consists in recording of oronasal airflow, respiratory effort signals and pulse oximetry^8^.

The apnea link (AL) polygraphy device is a recorder widely used in our setting^10-12^ in home-based diagnostic approaches. There are two different models (*Plus* and *Air*) featuring the same basic signals (i.e., airflow, respiratory effort and oximetry), which can be processed using the same type of software.

Although preliminary information suggests a high sensitivity (S) and specificity (Sp) of pulse oximetry in populations at a high risk for OSA, there is scarce published information comparing oxygen desaturation index (ODI) and AHI recordings obtained on the same night^13-14^. The use of oximetric data would allow patients to be prioritized on a waiting list and even treated in high-risk situations with a lower cost^13-17^.

Our hypothesis is that ODI allows reaching an OSA diagnosis in most patients using a home-based approach, while airflow and/or respiratory movements make only a minor contribution to ODI’s diagnostic capacity.

## OBJECTIVE

We design this analysis with the primary objective to assess the diagnostic capacity of oxygen desaturation index as compared to all signals in manual analysis of home-administered respiratory polygraphy.

## MATERIAL AND METHODS

### Design

This retrospective diagnostic accuracy study was conducted on the database of a community hospital. The protocol was approved by the Institutional Review Board pursuant to the Declaration of Helsinki, as amended (March 2018).

### Study population

**Figure 1:**
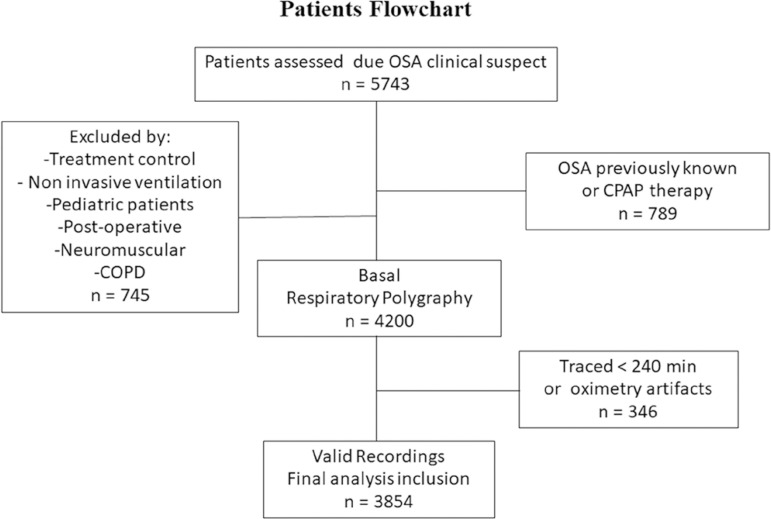
Flowchart patient’s selection.

The study population consists of consecutive patients >18 years old, examined between 2013 and 2018 (five-year period) due to suspected OSA. Exclusion criteria involved patients who needed oxygen therapy or non-invasive ventilation during RP and those with a known diagnosis of COPD, chronic heart failure, neuromuscular disease, insomnia, clinical symptoms of parasomnia, periodic limb movement disorder, clinical suspicion of narcolepsy, and less than 4 hours of valid recording time (Figure 1).

### Home-based self-administered respiratory polygraphy

Respiratory polygraphy recordings were obtained from patients referred to the sleep unit with a clinical suspicion of OSA based on one or more of the following cardinal signs: snoring, observed apnea or daytime sleepiness. Recordings were obtained using portable monitoring devices Apnea Link Plus and Air type III (ResMed, Sydney, Australia). Recorded signals included airflow measured by nasal pressure (linearized to obtain a flow-time curve), qualitative respiratory effort measured with thoracic band and pulse oximetry (SO_2_) with an average signal time of <1 second (XPod, Nonin, Plymouth, USA).

Patients were trained about the procedure by qualified hospital staff who showed them how to assemble, set up, and start polygraphs on the morning of the test day. Recordings were downloaded the following morning and analyzed manually (sequential manual edition) using Apnea Link® software 9.0 and 10.1 versions. Manual readings were performed by qualified pulmonologists in accordance with AASM guidelines (American Academy of Sleep Medicine)^8,9^.

### Measurements and definitions

Apnea was defined as a >90% drop in air flow, lasting more than 10 second^8,9^, and hypopnea as >50% and <90% drop in air flow, associated with a >3% drop in oxygen saturation - lasting more than 10 seconds (Chicago criteria)^8,9^.

AHI was calculated as the number of respiratory events (apneas + hypopneas) per hour of valid recording time. Recordings were classified either as normal (AHI <5 ev/h), mild (5-14.9 ev/h), moderate (15-29.9 ev/h), or severe (≥30 ev/h).

Oxygen desaturations ≥3% and ODI was automatically calculated (automatic algorithm) and classified using the same ODI cutoffs (ev/h).

### Statistical analysis

Variables were expressed according to their distribution either as standard deviation and mean values or as median values and 25-75% percentiles. Mann-Whitney or Chi-square tests were used to compare differences between false negative/positive results (FN)/(FP) and true positive/negative results (TP)/(TN). A *p*-value <0.05 was considered statistically significant. The area under the ROC curve, the S and Sp of ODI (study method) as compared to AHI (reference method) were estimated.

To quantify the reliability of the measurements associated with the continuous quantitative variables in the severity indicators (AHI and ODI), the intraclass correlation coefficient (ICC) was calculated.

Statistical analysis was conducted using Graph Pad Prism-7.04™ and MedCalc Statistical Software 19.1 (Bvba, Ostend, Belgium; http://www.medcalc.org).

## RESULTS

We included 3854 patients in our analysis. 61.5% were male. Age and body mass index were as follows: 55 years old (44-65) and 30.9kg/m^2^ (27-36).

Epworth sleepiness scale (ESS) and STOP-BANG questionnaire (median value and interquartile range) were: 7 points (4-11) and 4 components (3-6), respectively. 29% had ESS >10 points, 52% hypertension, and 57% were obese (BMI >30kg/m^2^).

Respiratory polygraphy indicators were (median value and interquartile range); AHI 14 events/hour (6-26), ODI 14.7 events/hour (7-27) and time spent with saturation <90% (T90), 7% (1-24%). 34% of patients were classified as having mild OSA, while 48% were classified as suffering from moderate to severe OSA. Patient characteristics are shown in Table 1.

**Table 1:** Characteristics of study population.

n=3854	Variables
**ClinicalCharacteristics**	
Age(years)*	55 (44-65)
Males	61.5%
Females	38.5%
BMI (kg/m^2^)*	30.9 (27-36)
-Obesity (BMI ≥30)	57%
Epworth score(points)*	7 (4-11)
-Epworth >10	29%
STOP-BANG score (SB)*	4 (3-6)
- SB ≥ 5	48.5%
Hypertension	52%
**Respiratory Polygraphy Indicators**	
Total Valid Recording Time(min)	396 (345-450)
AHI(events/hour)*	14 (6-26)
-AHI <5	18%
-AHI ≥5 y <15	34%
-AHI ≥15 y <30	27%
-AHI ≥30	21%
ODI (events/hour)*	14.7 (7-27)
T90 (%)*	7 (1-24)

* expressed as median value (interquartilerange); AHI: Apnea-hypopnea Index; ODI: O2 desaturation index ≥3%; T90: Time spent with oxygen saturation below90%.

S and Sp, positive/negative likelihood ratio (LR+/-) and ODI’s accuracy to diagnose OSA (AHI ≥5) were as follows; 93%, 92%, 12, 0.08 and 93% (Table 2). For an OSA defined as AHI ≥15, ODI performance was: S 94%, Sp 94%, RP+ 15, RP- 0.06 and 94% accuracy (See Table 2).

**Table 2:** Sensitivity (S), specificity (Sp), positive and negative likelihood ratio (LR) and predictive value (PV) of ODI as compared to AHI >5 events/hour.

ODI(events/hour)	S	CI95%	Sp	CI95%	LR+	CI95%	LR-	CI95%	PV+	PV-
>6,4 (AHI ≥5)	93	92-94	92	90-94	12	9-15	0,08	0,07-0,09	98	75
>7 (AHI ≥5)	90	89-91	95	93-97	18,38	13-25	0,11	0,10-0.1	99	68
>8 (AHI ≥5)	86	85-87	97	96-98	30,85	20-47	0,14	0,1-0.2	99	62
>9 (AHI ≥5)	82	81-83	99	97-99	58,73	32-109	0,18	0,2-0.2	100	56
>15,3 (AHI ≥15)	94	93-95	94	93-95	15	13-18	0,06	0,05-0.07	93	95
>16 (AHI ≥15)	92	90,5-93	95	94,5-96	20	17-25	0,09	0,07-0,1	95	93
>17 (AHI ≥15)	88	86-89	97	96-97,5	28	22-35	0,12	0,1-0,1	96	90
>18 (AHI ≥15)	84	82,5-86	98	97-98	36	27,5-48	0,16	0,1-0,2	97	87
>19 (AHI ≥15)	81	79-82.5	99	98-99	59	40,5-85	0,20	0,2-0,2	98	85
>6,4 (AHI ≥5 / <15)	84	82-86	92	90-94	11	8-13	0,17	0,2-0.2	95	76

**Figure 2:**
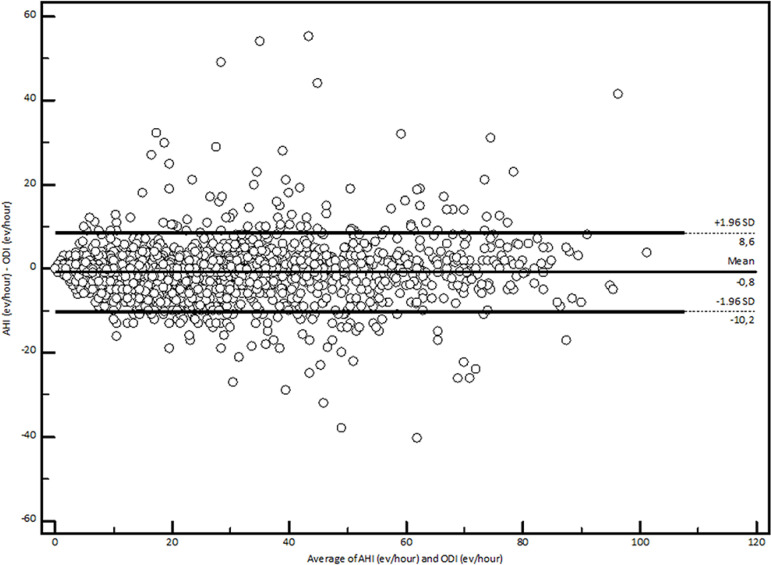
Correlation between ODI and AHI (ICC: 0.96, r^2^ = 0.92) in a Bland and Altman plot.

The mean difference between ODI and AHI was 0.8 (95% CI, 0.65-0.95) (Figure 2). The correlation (r^2^ Spearman) was 0.92 and the intraclass correlation coefficient (ICC) was 0.96 (total group), 0.83 for mild OSA and 0.93 for moderate to severe conditions.

Finally, we compare false negatives and positives (FN/FP) with true negative and positive (TN/TP) results. FN (n=219) had lower BMI as compared to TP (n=2919); 28 vs. 31.8kg/m^2^, *p*<0.001, and 96% of FN cases were classified as mild OSA patients with AHI in border range (median: 6. 25-75 interval: 5-7 events/hour). On the other hand, FP (n=57) had higher BMI (31 vs. 27kg/m^2^, *p*<0.001) and higher levels of hypoxemia (T90; 3% vs. 0%, *p*<0.001) and AHI similarly in border values (median: 4. 25-75 interval: 3-4 events/hour).

## DISCUSSION

Our results show that in a population at a high risk for OSA, the ODI calculated automatically with a self- administered home-based approach was strongly correlated with manually obtained AHI values (ICC 0.96, r^2^ 0.92). Likewise, an ODI >6 had an S, Sp, and accuracy >90% to diagnose OSA (AHI ≥5), with a LR+ >10 and a LR- <0.1. According to these results, pulse oximetry is a diagnostic tool with an acceptable performance in this clinical setting.

Randomized studies with appropriate statistical power have shown that simplified home-based tests are sufficient to diagnose patients at a high risk for moderate to severe OSA^15,16^. In our usual work environment, this is a widely used strategy^10-12^, which is included in international recommendations7-8 and national guidelines17. Additionally, as pulse oximetry is associated with lower direct costs, fewer logistic requirements, and shorter reading and interpretation times, some authors propose this test as initial screening for populations at risk for OSA^13^.

Inconsistent classifications between AHI and ODI were found in less obese patients who suffered from mild OSA, suggesting that oximetric indicators may be less useful to represent AHI in this specific group. In this regard, ODI’s consistency has been described in relation with higher BMI^11^. In addition, Fabius et al.^13^ describe a lower performance of ODI in older patients, hypothesizing that age-related comorbidities could act as possible confounders. These authors, who compared oximetric data and home-based RP results using an analytical method similar to ours, highlight the possible influence of population characteristics in the performance of indicators^13^. However, they conclude that the number of FN/FP cases seems acceptable (5%), which is consistent with our findings.

Currently, a new generation of algorithms to the oximetry signal is under development and its predictors could even improve the performance of traditional ODI. A recent study using data from Sleep Heart Health Study analyzed data derived from PSG due oximetry improved algorithms^18^ obtained a correct identification of those with IAH <15 events/hour with an accuracy of 87.6%.

A randomized controlled study conducted in Australia compared the efficiency of validated questionnaires and oximetry used by general practitioners working in rural areas to implement OSA treatment in patients with excessive daytime sleepiness. Their results were similar, in terms of improvement of symptoms, to those obtained by patients who underwent expert evaluation and conventional tests at a sleep unit. These findings open the door to new possible recommendations that could be interesting for remote areas or countries with limited resources^15^.

It is necessary to emphasize that our analysis was only focused on the performance of a measurement tool, since clinical decisions in sleep-disordered breathing are usually complex. In our country, a multicenter study of simulation of intention to treat based on clinical and oximetry, allowed sleep specialists to reliably indicate CPAP treatment for approximately 60% of the more symptomatic patients who required such therapy according to clinical criteria and PSG^19^.

Even though we evaluated overnight oximetry separately from other signals (airflow or respiratory effort), our study has some bias and related limitations. The contribution of effort signals seems modest in this context. However, it is important to point out that the oximetry traces from OSA patients cannot be differentiated from those of patients with central apnea, a field where RP provides particularly relevant information.

The criterion used for hypopnea calls for a deeper reflection, both in our study and similar ones^8-9,11-13^. The AASM has updated the definition of hypopnea with a more sensitive cutoff (reduction of airflow signal by >30%) and an associated desaturation of 3%^9^. Even though we used the same desaturation criterion for expert and software analysis, the comparison between both definitions of hypopnea results in classification changes in up to 60% of events - which may impact the level of consistency of different indicators^20^. At present, we are using the Chicago Criterion (1999), which is based on a >50% and <90% drop in flow.

The study population imposes another limitation, since patients were selected at a specialized center that provides treatment to OSA patients who show high-risk indicators (STOP-BANG, Epworth, Hypertension, degree of obesity, etc.). Oximetry could perform differently in the general population.

Our study does not strictly represent a validation test because both metrics were taken with the same instrument, and that the ODI and AHI indicators share part of the definition, which could modify the interpretation of our results.

Furthermore, we use cutoff values for ODI classification used in other similar studies^10-13^, even when its clinical utility and pathophysiological significance is not identical to that of AHI.

Finally, our study analyzed the performance of an automatic scoring algorithm that belongs to a specific polygraph model and, therefore, our results cannot be extrapolated to other devices.

## CONCLUSION

In a population at a high risk for OSA, home-based pulse oximetry had a diagnostic accuracy >90% when is compared against all respiratory signals obtained from simplified home sleep testing. Respiratory flow events permitted the diagnosis of an additional 10% of subjects with obstructive sleep apnea, especially patients with mild presentations who were not correctly classified by oxygen desaturation index.
